# Efficacy of mistletoe extract as a complement to standard treatment in advanced pancreatic cancer: study protocol for a multicentre, parallel group, double-blind, randomised, placebo-controlled clinical trial (MISTRAL)

**DOI:** 10.1186/s13063-020-04581-y

**Published:** 2020-09-11

**Authors:** Kathrin Wode, Johanna Hök Nordberg, Gunver S. Kienle, Nils O. Elander, Britt-Marie Bernhardson, Berit Sunde, Lena Sharp, Roger Henriksson, Per Fransson

**Affiliations:** 1 Department Nursing, Umeå University, Umeå, Sweden; 2 Department Radiation Sciences, Umeå University, Umeå, Sweden; 3 Department Upper Abdominal Diseases, Karolinska University Hospital, Stockholm, Sweden; 4 Regional Cancer Centre Stockholm Gotland, Stockholm, Sweden; 5 Department Neurobiology, Caring Sciences, Society and Department Physiology and Pharmacology, Karolinska Institutet, Stockholm, Sweden; 6 Center for Complementary Medicine; Institute for Infection Prevention and Hospital Epidemiology, Medical Center, University of Freiburg, Faculty of Medicine, University of Freiburg, Freiburg, Germany; 7 Institute for Applied Epistemology and Medical Mehodology at the University Witten/Herdecke, Freiburg, Germany; 8 Department Oncology and Department Biomedical and Clinical Sciences, Linköping University, Linköping, Sweden; 9 Department Learning, Informatics, Management, and Ethics, Karolinska Institutet, Stockholm, Sweden; 10 Department Clinical Intervention and Technology, Division Surgery, Karolinska Institutet, Stockholm, Sweden; 11 Cancercentrum, Norrland University Hospital, Umeå, Sweden

**Keywords:** Neoplasms, Pancreatic neoplasms, Mistletoe, Complementary therapies, Palliative care, Quality of life, Clinical trial, Randomised controlled trial

## Abstract

**Background:**

Most pancreatic cancer patients present with advanced stage at diagnosis with extremely short expected survival and few treatment options. A multimodal palliative approach is necessary for symptom relief and optimisation of health-related quality of life. In a recent open-label trial of mistletoe extract for advanced pancreatic cancer patients not eligible for chemotherapy, promising results on improved overall survival and better health-related quality of life were reported.

The objective of the present study is to assess the value of mistletoe extract as a complement to standard treatment (palliative chemotherapy or best supportive care) in advanced pancreatic cancer patients with regard to overall survival and health-related quality of life.

**Methods:**

The trial is prospective, randomised, double-blind, multicentre, parallel group and placebo-controlled. In total, 290 participants are randomly assigned to placebo or mistletoe extract given subcutaneously in increasing dosage from 0.01 to 20 mg three times per week for 9 months. Stratification is performed for site and palliative chemotherapy. Main inclusion criteria are advanced pancreatic cancer and Eastern Cooperative Oncology Group performance status 0 to 2; main exclusion criteria are life expectancy less than 4 weeks and neuroendocrine tumour of the pancreas. Two ancillary studies on sub-sets of participants are nested in the trial: a biomarker study collecting blood samples and a cross-sectional qualitative study with semi-structured face-to-face interviews.

**Discussion:**

To our knowledge, this is the first placebo-controlled randomised trial assessing the impact of mistletoe extract as a complement to standard treatment on overall survival and health-related quality of life in patients with advanced pancreatic cancer. The presented trial with its two nested ancillary studies exploring biomarkers and patient experiences is expected to give new insights into the treatment of advanced pancreatic cancer.

**Trial registration:**

EU Clinical Trial Register, EudraCT Number 2014-004552-64. Registered on 19 January 2016.

ClinicalTrials.gov NCT02948309. Registered on 28 October 2016.

## Administrative information

Note: the numbers in curly brackets in this protocol refer to SPIRIT checklist item numbers. The order of the items has been modified to group similar items (see http://www.equator-network.org/reporting-guidelines/spirit-2013-statement-defining-standard-protocol-items-for-clinical-trials/).

**Table:** 

Title {1}	Efficacy of mistletoe extract as a complement to standard treatment in advanced pancreatic cancer: study protocol for a multicentre, parallel group, double-blind, randomised, placebo-controlled clinical trial (MISTRAL)
Trial registration {2a and 2b}	EU Clinical Trial Register, EudraCT Number 2014-004552-64. Registered 19 January 2016 ClinicalTrials.gov, Identifier: NCT02948309. Registered 28 October 2016
Protocol version {3}	Date 15 July 2020, Version 3.3
Funding {4}	The trial is academic, with financial support provided by grants from the Oncological Department Endowment Fund at Karolinska University Hospital, The Cancer Research Funds of Radiumhemmet, Gyllenberg foundation, Ekhaga foundation, Dagmar Ferbs Memorial fund, Cancer Research Foundation in Northern Sweden and The Sjöberg Foundation. The funders did not have any role in trial design nor writing of the study protocol or this paper. No commercial interests are involved in the trial. Regional Cancer Centre Stockholm Gotland provided support with administration, research time for coordinating investigator, monitoring and analytic competence. Iscador AG, Switzerland manufacture and supply both mistletoe extract and placebo free of charge.
Author details {5a}	KW: Dept. Nursing and Dept. Radiation Sciences, Umeå University, Umeå; Dept. Upper Abdominal Diseases, Karolinska University Hospital and Regional Cancer Centre Stockholm Gotland, Stockholm, Sweden JHN: Regional Cancer Centre Stockholm Gotland and Dept. Neurobiology, Caring Sciences, Society and Dept. Physiology and Pharmacology, Karolinska Institutet, Stockholm, Sweden GSK: Centre for Complementary Medicine; Institute for Infection Prevention and Hospital Epidemiology, Medicine and Medical Centre, Faculty of Medicine, University Freiburg and Institute for Applied Epistemology and Medical Methodology at the University Witten/Herdecke, Freiburg, Germany NOE: Dept. Oncology and Dept. Biomedical and Clinical Sciences, Linköping University, Linköping, Sweden BMB: Dept. Learning, Informatics, Management, and Ethics, Karolinska Institutet, Stockholm, Sweden BS: Dept. Nursing, Umeå University, Umeå; Dept. Clinical Intervention and Technology, Div. Surgery, Karolinska Institutet and Dept. Upper Abdominal Diseases, Karolinska University Hospital, Stockholm, Sweden LS: Regional Cancer Centre Stockholm Gotland and Dept. Learning, Informatics, Management and Ethics, Karolinska Institutet, Stockholm, Sweden RH: Dept. Radiation Sciences, Umeå University, and Cancercentrum, Norrland University Hospital, Umeå, Sweden PF: Dept. Nursing, Umeå University, and Cancercentrum, Norrland University Hospital, Umeå, Sweden
Name and contact information for the trial sponsor {5b}	Dept. Upper Abdominal Diseases, Karolinska University Hospital, Stockholm, Sweden Contact and coordinating investigator Kathrin Wode, kathrin.wode@sll.se, phone: +46 8 123 142 61
Role of sponsor {5c}	The sponsor is non-commercial and represents the main study site, where most participants are expected to be recruited due to its size. The sponsor supports the study with trial unit facilities and study nurses. Karolinska University Hospital is responsible of personal data according to the General Data Protection Regulation (GDPR).

## Introduction

### Background and rationale {6a}

Pancreatic cancer is currently the fourth most common cause of cancer-related deaths worldwide and is estimated to climb to second place by 2030 [1]. Despite improved treatment strategies, the prognosis remains extremely poor. The combined impact of severe symptoms and comorbidities associated with the disease most often lead to a rapid deterioration of performance status and health-related quality of life (HRQoL). According to a systematic review on real-world data from Europe, the disease causes an almost complete loss of healthy life [2]. One-year survival for all stages is about 21% and 5-year survival about 9% [3]. The only option for cure is surgical resection, preferably followed by 6 months of postoperative combination chemotherapy [4, 5]. Unfortunately, 80–85% of the newly diagnosed patients present with locally advanced and/or metastatic disease which preclude this type of curative intent strategy. In addition, most of patients undergoing curative intent surgery will relapse in 2 to 3 years [6].

For patients with primary unresectable disease, or recurrent disease under the post-resection follow-up, survival time is usually short. In these groups, a multimodal palliative approach is necessary to relieve cancer-related symptoms such as nausea, loss of appetite and weight, cachexia, and fatigue, and to optimise the HRQoL. In patients with adequate Eastern Cooperative Oncology Group (ECOG) performance status 0 to 2 and acceptable organ functions, palliative chemotherapy may prolong life and reduce disease-related symptom burden. For reasonably ‘fit’ patients treated with chemotherapy combination regimens such as gemcitabine/nab-paclitaxel or FOLFIRINOX, overall survival is usually between 8 and 11 months [7–10]. For patients with lower performance status and comorbidity, best supportive care or palliative chemotherapy with milder regimens such as gemcitabine monotherapy remain the only therapeutic options. In these groups of patients, survival is usually limited to around 1–2 [9] and 6 months respectively, the latter which appears similar in randomised controlled trials and real-world populations [7–9, 11].

Palliative supportive care, both as in- and outpatient care, is publicly funded and available to all patients in Sweden regardless of socioeconomic status.

Mistletoe (*Viscum album* L.) extract (ME) is widely used in integrative cancer care treatments, particularly in Europe [12–14]. *Viscum album* L. is a hemiparasitic shrub, growing on different host trees. Several pharmacologically active compounds have been isolated, such as mistletoe lectins I, II and III [15], viscotoxins [16, 17], oligo- and polysaccharides [18, 19], lipophilic extracts [20], triterpenes [21, 22] and others [23, 24]. The most prominent properties of ME are their cytotoxic and growth-inhibiting effects, which have been demonstrated in a variety of human tumour cell lines, lymphocytes and fibroblasts in vitro [23, 24]. The cytotoxic effects are mainly due to the apoptosis-inducing mistletoe lectins, while the viscotoxins induce necrotic cell death [23–25]. ME are also recognised for their immune-modulating activity: activation of monocytes/macrophages, granulocytes, natural killer (NK) cells, NK cell-mediated tumour cell lysis, T cells (especially T helper cells), boost of T cell-mediated killing and induction of various cytokines [23, 24, 26, 27]. Further, components of ME also downregulate the expression of tumour genes, reduce motility and invasiveness of tumour cells [26] and show antiangiogenetic effects [28]. They also reduce chromosome damage and improve endogenous DNA repair mechanisms [23, 24, 29, 30]. In animals, ME display anti-tumour effects when administered either directly into the tumour or systemically [23, 24, 27, 31].

Clinical effectiveness of ME in cancer has been investigated in many studies with various designs and methodological quality—among these, more than 40 prospective randomised controlled trials [23, 31–39]. With regard to study quality and consistency of results, the best evidence exists for improvement of HRQoL and enhanced tolerability of cytoreductive therapies [32, 33, 35]. Regarding survival, a randomised controlled Serbian trial including 220 patients with advanced pancreatic cancer not eligible for palliative chemotherapy reported a median survival of 4.8 months among ME-treated patients compared with 2.7 months in the control group (HR = 0.49; 95% CI = 0.36–0.65; *p* < 0.0001). The survival benefit of ME was larger among patients with good prognostic features (6.6 vs. 3.2 months) than with poor prognostic features (3.4 months vs. 2.0 months) [36]. In addition, patients reported substantially better HRQoL and tended to gain weight when treated with ME compared to the control group [35]. Two phase I/II trials and some retrospective studies also reported favourable outcomes regarding safety and efficacy of ME in patients with pancreatic cancer [40–42]. Tumour remission under ME is rare but has been reported in some small studies and case reports, mostly applying high-dose ME directly at the tumour site [31, 43–46]. Ongoing trials investigate ME in bladder cancer (NCT02106572) and other advanced solid tumours (NCT03051477). ME are considered safe and well tolerated even among immunosuppressed patients. However, in rare cases, a generalised allergic reaction may occur [23, 24, 47, 48]. Unwanted and/or clinically significant interactions with ME and established chemotherapeutic drugs have not been observed [23, 40, 49–52].

### Objectives {7}

The overall objective of the present trial with two ancillary studies is to assess the value of ME as a complement to standard treatment in advanced pancreatic cancer.

The primary objective is to compare overall survival (OS) in patients with advanced pancreatic cancer randomised to either ME or placebo.

The secondary objectives are to compare health-related quality of life (HRQoL), body weight, corticosteroid use, adverse events (AE) and costs for supportive care and inpatient care.

Ancillary studies

Two ancillary studies aim to

1. Assess immunological effects and to explore potential prognostic and predictive biomarkers (biomarker study).
2. Explore advanced pancreatic cancer patients’ experiences of every-day life (qualitative study)

### Trial design {8}

The study is designed as a phase III prospective, randomised, double-blind, multicentre, parallel group, placebo-controlled clinical trial. A total of 290 participants are randomly assigned to receive ME or placebo in a one-to-one ratio, stratified by site and palliative chemotherapy (eligible or not). Superiority testing will be used.

Ancillary studies

Two ancillary studies on sub-sets of participants are nested in the trial:

1. A biomarker study collecting blood samples
2. A cross-sectional qualitative study with semi-structured face-to-face interviews

## Methods: participants, interventions and outcomes

### Study setting {9}

The trial is an academic multicentre study and started with a limited number of study sites due to organisational reasons. Subsequently, additional study sites have been amended to reach target inclusion as fast as possible. Currently, nine qualified study sites in Sweden are participating: community hospitals in Västerås (Västmanlands Hospital), Jönköping (Ryhov County Hospital), Skövde and Lidköping (Skaraborg Hospital), Karlstad (Central Hospital Karlstad), Kalmar (Kalmar County Hospital) and the South General Hospital in Stockholm, as well as the University hospitals in Umeå (University Hospital of Umeå), Linköping (University Hospital of Linköping) and Stockholm (Karolinska University Hospital).

Ancillary studies

1. Current study sites collecting data for the biomarker study: South General Hospital in Stockholm, University Hospital of Linköping and Karolinska University Hospital
2. Study sites collecting data for the qualitative study: Västmanlands Hospital, Karolinska University Hospital, South General Hospital and University Hospital of Linköping

### Eligibility criteria {10}

#### Inclusion and exclusion criteria for participants

Patients eligible for inclusion in the trial and the two ancillary studies must meet all the following criteria:

- Signed written informed consent
- Age ≥ 18 years
- Inoperable locally advanced or metastatic pancreatic cancer or relapse of pancreatic cancer
- Primary diagnosis: if histology is not clinically achievable, diagnosis is to be confirmed according to local practice sufficient for diagnosis and choice of therapy (such as CA19-9 and CT)
- Relapse: histology (not required) or diagnosis according to local practice such as clinical signs and/or imaging and/or CA19-9
- ECOG performance status 0–2
- Adequate negative pregnancy test and adequate contraception (where appropriate)

Exclusion criteria

- Life expectancy less than 4 weeks
- Pregnancy or breastfeeding
- Neuroendocrine tumours of the pancreas
- Current use of interferon, granulocyte-colony stimulating factor and thymus preparations
- Symptomatic brain oedema due to brain metastases
- Known hypersensitivity to mistletoe-containing products
- Current use of ME preparations in any form
- Chronic granulomatous disease or active autoimmune disease or autoimmune disease with immunosuppressive treatment
- Medical, psychiatric, cognitive or other conditions that may compromise the patient’s ability to understand the patient information, give informed consent, comply with the study protocol or complete the study (e.g. needle phobia)

#### Study site requirements

Centre selection is based on the presence of appropriate clinical and research infrastructure (research unit with study nurses) and principle investigators (oncologists) with Good Clinical Practice (GCP) qualifications.

Ancillary studies

1. The biomarker study is being conducted at some of the centres where biobanking and sample collection was organizationally possible
2. The qualitative study is being conducted at four centres representing urban, rural and small-town environments for purposeful sampling

### Who will take informed consent? {26a}

It is each investigator’s responsibility to give adequate oral and written information on the study’s purpose and procedures, information on data protection procedures, possible advantages and disadvantages of participation, and option to withdraw from the study at any time and without any given reason. Written informed consent must be obtained for all participants prior to any trial-related procedures.

### Additional consent provisions for collection and use of participant data and biological specimens {26b}

For the ancillary studies, separate information sheets are provided, and written informed consent must be signed prior to

1. Any collection of blood samples in the biomarker study and
2. Prior to face-to-face interviews in the qualitative study

## Interventions

### Explanation for the choice of comparators {6b}

Isotonic saline solution was chosen as a placebo to avoid any possible harm from injections and because it is the vehicle of used ME.

### Intervention description {11a}

The study intervention consists of subcutaneous injections with a herbal fermented aqueous extract of *Viscum album* L (Santalaceae; European mistletoe) grown on *Quercus* (Fagaceae; oak tree). The product used is Iscador® Qu, manufactured by Iscador AG, Switzerland. The product is registered as a herbal medicinal product for well-established use in Sweden. The drug substance is a fermented aqueous extract of fresh mistletoe (drug-to-finished extract ratio 1:5) mixed with water and sodium chloride to achieve an isotonic solution.

Composition of 1 mg ampoule:

**Table:** 

Product	Quantity of drug substance per 1 mg ampoule; extract 1:5	Corresponding amount of fresh mistletoe
Iscador® Qu 0.01 mg	0.05 mg	0.01 mg
Iscador® Qu 0.1 mg	0.5 mg	0.1 mg
Iscador® Qu 1 mg	5 mg	1 mg
Iscador® Qu 10 mg	50 mg	10 mg
Iscador® Qu 20 mg	100 mg	20 mg

Mistletoe species and host tree are identified visually by a botanist. Extracts are characterised by a chromatographic identity test and have a specified content of viscotoxins as marker substances. Retained samples of active ingredient and the study medication are kept at the manufacturer headquarters of Iscador AG, Switzerland.

Participants (or their next of kin) are instructed in injection technique (injection speed 20–30 s, injection site abdominal wall or, if not possible, proximal thigh). Treatment is given with one ampoule (1 ml) 3 days per week (Monday–Wednesday–Friday or Tuesday–Thursday–Saturday), preferably in the morning on chemotherapy-free days to minimise the risk of potentially confounding side effects of chemotherapy. The dose is gradually increased, starting with two 0.01 mg injections over four 0.1-mg, four 1.0-mg and four 10-mg injections, followed by the highest possible dose of 20 mg as the maintenance dose for the rest of the trial. Participants receive boxes with the study drug in 1-ml ampoules in different colour-coded concentrations.

### Criteria for discontinuing or modifying allocated interventions {11b}

Study drug application and dose increase may be modified as below for the following reasons:

#### Maintenance of dose

According to traditional clinical use, occurrence of local reactions (defined as redness < 5 cm, itching, warmth, swelling within hours after injection and ceasing within 48 h) leads to maintenance of the actual dose until no further local reaction has occurred for 1 week; then, outlined dose increase is continued.

#### Reduction of dose

The dose is reduced if a local *over*reaction (defined as local reaction with redness > 5 cm and/or remaining for > 48 h) or malaise or flu-like symptoms according to the Common Terminology Criteria for Adverse Events (CTCAE) [53] grade ≥ 2 or fever > 38 °C (not caused by infection or tumour) with clear time correlation to dose increase and treatment days are observed. Half (=0.5 ml) of the ampoule content of the symptom-causing dose will then be used for 2 weeks, and subsequently the former symptom-causing dose, followed by the planned dose increase are given. If the symptoms recur, participants stay at the lower dose. If fever occurs due to the trial intervention, antipyretics are dissuaded, except for analgesic purposes. If an activation of local chronic inflammatory processes is observed (a very rare side-effect of ME), dosage is decreased and adapted individually.

#### Temporal interruption of trial intervention

The study treatment will be temporarily interrupted in case of acute infectious disease with fever > 38 °C and/or clinical symptoms from a clinically relevant infection. Upon clinical recovery, treatment continues with the next lower dose and the dose is increased as planned.

#### Termination of trial intervention

Study treatment is terminated after 9 months from the baseline visit. Treatment may be terminated earlier due to the participant’s own decision to withdraw consent or in case of medical, psychiatric, cognitive or other conditions occurring that may endanger the patient’s ability to comply with study protocol or complete the trial. Other reasons for early treatment termination are serious adverse events with suspected causal relationship to the study drug (such as allergic reaction CTCAE Grade ≥ 2, anaphylaxis, erythema multiforme or other events which may cause severe or permanent harm), or major violation of study protocol. The treatment might also be stopped in the very late palliative stage, if a potentially life-extending effect is no longer considered desirable.

### Strategies to improve adherence to interventions {11c}

#### Drug account

**Fig. 1:**
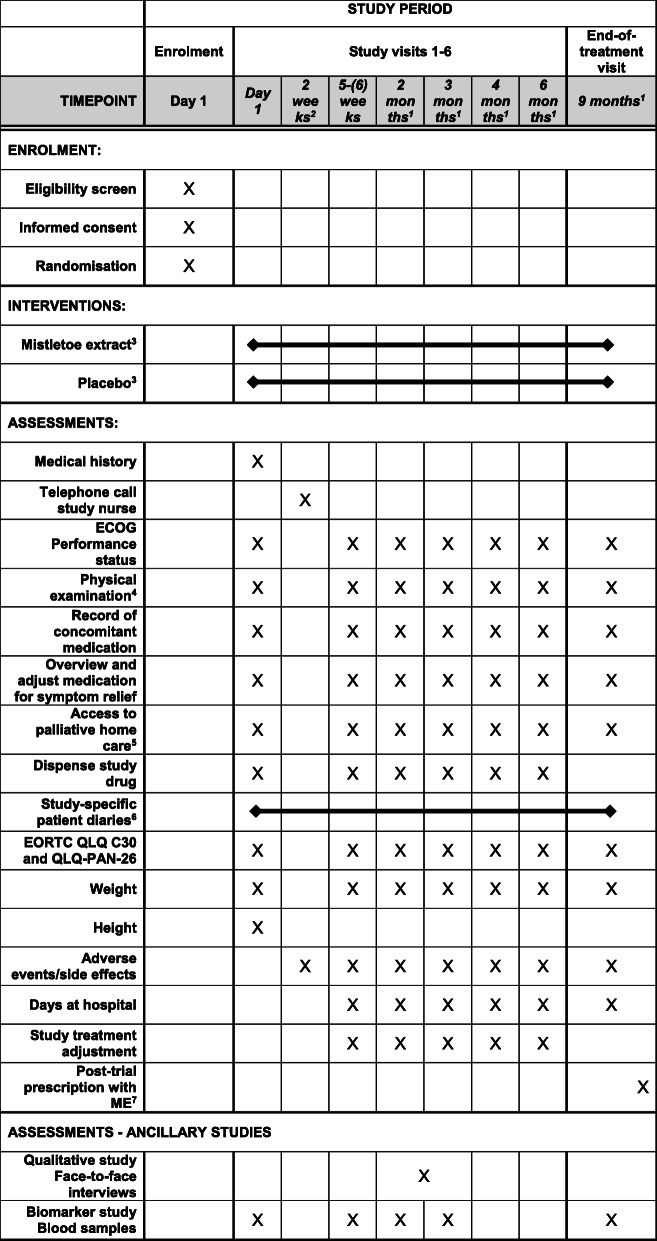
Schedule of enrolment, interventions and assessments ^1^ Or as close to this date as possible ^2^ Phone call by study nurse ^3^ Three subcutaneous injections per week with placebo/ME; dose escalation from 0.01mg to 20mg ^4^ At screening visit: general condition, heart, lungs, abdomen and icterus (yes/no). At subsequent visits at least abdomen, icterus (yes/no) and general condition ^5^ Access is documented; referral if medical need for access ^6^ Recorded by participants in study-specific patient diary: n total parenteral nutrition infusions per week, n visits from palliative home care team per week, consumption of n dietitian-prescribed nutritional supplement drinks per day, injections, dosage, interruptions of study treatment, comments ^7^ Voluntary for participants at end of study

At each visit, intervention dosage according to the protocol is prescribed and documented in the study-specific patient diary, where participants also keep a record of the administered injections. Participants are dispensed as much study drug as needed until the next scheduled visit, plus extra supplies to cover for potential dose modifications or change of visits for any reason. To evaluate adherence to treatment, participants return their completed diary at each follow-up visit and receive a new diary covering the time until next outlined visit (Fig. 1).

#### Education study sites, palliative home care teams

Study sites and related palliative home care teams are instructed on study procedures at start meetings and subsequently based on local needs during the trial. Annual national trial meetings for study personnel and investigators are held, and a periodical newsletter is sent for updates on the state of the study.

### Relevant concomitant care permitted or prohibited during the trial {11d}

Through the Swedish health care system, participants have access to palliative supportive care, either as in- or outpatient care, for symptom relief and psychological and social support.

Initiation or termination of or switch to a new line of palliative chemotherapy does not affect the patients’ participation in the trial. Palliative radiotherapy is permitted.

As no drug-drug interactions with ME are known, all drugs, except those mentioned in exclusion criteria, are allowed. Each drug given during study participation is regarded as concomitant medication and is registered in the electronic case report form (eCRF), including complementary compounds such as nutritional supplements, vitamins, natural remedies and homoeopathic drugs.

### Provisions for post-trial care {30}

Trial participants are covered by Swedish Pharmaceutical Insurance. Before study entry, potential participants are informed via the consent form that they will be offered post-trial treatment with ME for free.

### Outcomes {12}

Primary endpoint for the trial is OS defined as time from randomisation to death of any cause.

Secondary endpoints are HRQoL measured by the European Organisation for Research and Treatment of Cancer (EORTC) HRQoL questionnaires QLQ-C30 [54] and QLQ-PAN-26 [55] at study visits (baseline, 5–6 weeks, 2, 3, 4, 6 and 9 months after randomisation), body weight (documented weekly in patients’ study-specific diaries and at abovementioned study visits), corticosteroid use (continually from participants’ health records from baseline visit to end of study participation), adverse events (assessed at abovementioned study visits and continually from health records from baseline visit to end of study participation), costs for supportive care and costs for inpatient care (calculated from documentation in study-specific patient diaries and continually from health records from baseline visit to end of study participation).

OS as primary endpoint and HRQoL as secondary endpoints are chosen due to their high clinical relevance in pancreatic cancer patients, reflected by the fact that all palliative oncological treatment in this patient group aims to both improve HRQoL and to prolong survival, if possible.

Ancillary studies

1. Endpoints include analyses of blood cell count, differential, leukocyte subtypes, CRP, CA 19-9, albumin, anti-mistletoe lectin antibodies, IgG subtypes and cytokines at baseline and under follow-up. Whole blood is collected at baseline for isolation of DNA from peripheral leukocytes and subsequent analyses of DNA sequence and variations.
2. The outcome of the qualitative study is to understand participants’ every-day life situations, symptom burden and management, self-administration of subcutaneous injections and experiences of participating in a randomised placebo-controlled trial on ME.

### Participant timeline {13}

The schedule of enrolment, interventions and assessments (Fig. 1) illustrates trial procedures from enrolment to end of study.

### Sample size {14}

Calculation of sample size was based on a number of considerations based on comparability with the randomised controlled trial on ME in advanced pancreatic cancer conducted in Serbia [36]. A basic assumption was that OS data from the control group in Serbia were comparable to OS data from Swedish patients not eligible for palliative chemotherapy. This assumption was supported by available quality assessments on survival in this type of patients undergoing palliative supportive care in Stockholm (unpublished data). Moreover, HR was estimated to be affected by longer OS for patients eligible for palliative chemotherapy due to better prognosis in general, expected higher proportion of participants in strata for palliative chemotherapy than in strata for best supportive care, potentially longer OS in control groups in both strata due to extensive palliative care [56] and expected longer time from diagnosis to trial inclusion than in Serbia. Finally, pragmatic reasons affecting feasibility (academic trial, comparatively rare type of cancer, trial duration) were considered.

A two-sided log rank test with an overall sample size of 290 patients (145 in the placebo group and 145 in the ME group) achieves 90% power at a 5% significance level to detect a hazard ratio of 0.67. In the placebo group, survival time in patients not eligible for palliative chemotherapy is expected to be shorter than for patients receiving palliative chemotherapy. However, the effect size for patients receiving ME compared to patients in the placebo group with respect to overall survival is expected to be the same in the two strata, i.e. a hazard ratio of 0.67.

Ancillary studies

1. Within the biomarker study, blood samples from at least 60 patients will be collected
2. Within the qualitative study, approximately 30 participants from both the intervention and placebo groups will be interviewed

### Recruitment {15}

As patients get their primary diagnosis or relapse in different contexts, potential participants are identified at multidisciplinary conferences and/or at therapy conferences at associated surgical departments, at the oncology departments of the study centres or in associated palliative home care units.

Ancillary studies

1. For the biomarker study, participants are recruited consecutively at baseline visit.
2. For the qualitative study, participants are recruited in-between visit three and four by phone-call from the qualitative research team. Sample heterogeneity is strived for by inclusion of an equal distribution from interventional and control groups, both women and men, with variation in ages and geography (city/countryside) and treatment (best supportive care/palliative chemotherapy).

## Assignment of interventions: allocation

### Sequence generation {16a}

Participants are randomly assigned to either interventional or control group with a one-to-one allocation as per computer-generated randomisation in the Dynareg system [57]. Stratification is performed by site and eligibility for palliative chemotherapy (eligible or not). Block randomisation is used; block size is not disclosed to ensure concealment.

### Concealment mechanism {16b}

Participants are randomised using an online, central computerised randomisation system within the Dynareg system based on Microsoft ASP.NET in combination with Microsoft SQL-Server. The system is designed to support GCP-compliant data management [57]. Allocation concealment is insured as the service will not release the randomisation code until the patient has been recruited into the trial.

### Implementation {16c}

All patients who give consent for participation and fulfil the inclusion criteria are enrolled at study sites by investigators. They are registered by a unique code and randomised online by a staff member (investigator or study nurse); the randomisation system creates a code identical with batch numbers on study drug boxes at the study site. The number of codes and allocation to study arm are concealed to all study personnel apart from the data manger.

## Assignment of interventions: blinding

### Who will be blinded {17a}

All study personnel, care providers and trial participants, apart from the data manager (who has no contact with participants), are blinded for treatment allocation until end of the trial.

Blinding for study drug is achieved by ensuring identical appearance, shape, labelling and packaging of the study drug and placebo.

### Procedure for unblinding if needed {17b}

There should not be any need to unblind the allocated treatment as there is no antidote to ME. Nevertheless, unblinding can be carried out by the study site investigators and study nurses online in the randomisation system and would then be logged and clearly visible in the system.

## Data collection and management

### Plans for assessment and collection of outcomes {18a}

#### Primary outcome

Overall survival is primary outcome. 

After all included participants have fulfilled their treatment period (*n* = 290), survival status and eventual date of death will be checked for all participants at the Swedish Tax Agency’s register covering all inhabitants in Sweden and updated on a daily basis.

#### Secondary outcomes

We will measure generic and disease-specific aspects of *HRQoL* using the following validated questionnaires at baseline, 6 weeks and 2, 3, 4, 6 and 9 months after randomisation:

- EORTC QLQ-C30 [54], a generic questionnaire developed to assess HRQoL of patients with cancer
- EORTC QLQ-PAN26 [55], a questionnaire developed to assess the HRQoL for patients with pancreatic cancer

*Body weight* will be assessed at every visit and participants will also report weekly weight measures via the study-specific patient diary.

*Corticosteroid use* is assessed by recording concomitant medication at every visit.

*Adverse events* will be assessed at every scheduled visit, via telephone contact and at unscheduled visits.

Data for calculation of *observed costs* for supportive care and inpatient care will be collected at every visit by assessment of concomitant medication and days in hospital since previous visit. In addition, study-specific patient diaries provide information on need of total parenteral nutrition, visits from the palliative home care team and consumption of dietitian-prescribed nutritional supplement drinks.

*Baseline information* on diagnosis, such as imaging, histology, tumour classification and potential previous treatment such as surgery and chemotherapy for participants with relapse, will be collected from participants’ medical records. Medical history is taken at baseline visit. Data from telephone contacts and unscheduled visits will be collected continuously.

Data on ECOG *performance status*, *physical examination*, *concomitant medication*, *access to* (and if needed referral to) *palliative home care team*, *body weight* (and *height* at baseline visit), *spontaneously reported local reactions* and *side effects* will be collected at each study visit. The ECOG scale is used for scoring of performance status. The numbering scale from 0 (fully active) to 4 (completely disabled) helps to assess the functional status of a patient in terms of their ability to care for themselves, daily activity and physical ability (walking, working, etc.).

*Drug account*, *compliance* and *changes or interruptions in study treatment* will be collected from study-specific patient diaries covering the time between study visits and if necessary, completed by inquiry of the participant or their next of kin.

Ancillary studies

1. Blood samples from participants in the biomarker study are collected at visits 1, 2, 3, 4 and 7.
2. Semi-structured face-to-face interviews with participants in the qualitative study will be conducted in month 2 to 3. The semi-structured interview guide includes questions about patients’ experience of every-day life, symptom burden and management, self-administration of subcutaneous injections, and participation in the trial

#### Documentation and training plans

The eCRF is the main document for data collection, with study personnel filling in the required information from study visits and study-specific patient diaries, as well as collected HRQoL questionnaires.

Each centre’s personnel are introduced to the study protocol and requirements at a trial start meeting. Participating centres are trial units at oncological and surgical departments in publicly funded hospitals, ensuring high quality of study-specific procedures and data collection.

Ancillary studies

1. Blood samples will be collected by health care professionals according to national requirements. The samples are either analysed immediately at accredited laboratories according to clinically validated protocols, or aliquoted and frozen at − 80 °C for long-term storage. Biobanking is performed according to national regulations and guidelines at accredited biobank facilities at two of the academic sites (Stockholm and Linköping).
2. Interviewers in the qualitative study are trained and supervised by the principle investigator. The four interviewers will meet regularly during the interview period to discuss and calibrate interview technique and findings. If necessary, the interview guide will be adjusted, as is common in qualitative approaches, [58] to obtain rich data. The interviews are recorded and transcribed verbatim, with ongoing analysis throughout the interview process using the computer software NVivo 11

### Plans to promote participant retention and complete follow-up {18b}

As most participants in the trial are severely ill with limited physical strength, and each visit to the hospital may have a negative impact on HRQoL, efforts are made to minimise the number of visits by combining study visits with planned visits for oncological assessment during chemotherapy whenever possible. Follow-up visits in hospital may even be replaced by home visits, involving palliative home care teams, or by phone calls, if the patient is not able to come for a visit, e.g. due to poor physical condition. Logistics related to study-specific patient diaries, study drug supply and questionnaires are dealt with at the study site according to local routines.

Ancillary studies

1. For the biomarker study, blood sample collections are (as far as possible) coordinated with other blood tests for oncological treatment to avoid patient discomfort and inconvenience.
2. In the qualitative study, participants may choose a time and place for interview.

### Data management {19}

Data are collected from electronic medical records at the study sites, participants’ study-specific diaries, HRQoL questionnaires and trial-specific checklists. All collected data for the trial is entered electronically in eCRFs in the online Dynareg system, designed to support GCP-compliant data management [57]. The Dynareg System is based on Microsoft ASP.NET in combination with Microsoft SQL-Server. All traffic is encrypted with Secure Sockets Layer (SSL/HTTP) and cannot be accessed by a third party. User accounts are personal and all attempts to log into the system are logged. The system restricts what data and functionality a specific user has access to depending on the user’s organisation and role. All data changes are logged by user and time and are thereby traceable. Participants are registered with their patient study ID.

For most of the collected data, data entry is performed at participating study sites by both investigators and study nurses, and for some data such as HRQoL questionnaires and biochemistry, at the Regional Cancer Centre Stockholm Gotland as a central site. The option to choose a value from a list is available where applicable. The system checks that all registered data is of the correct type—dates must be valid dates and numeric numbers must be valid numbers, etc. Functionality for logical checks/validation, missing data and specific errors is used where appropriate in the eCRF.

Ancillary studies

1. Data from the biomarker study is recorded under study-specific codes according to national and European regulations. Neither laboratory personnel nor unauthorised co-workers will have any access to the study specific code key.
2. Data from the qualitative study, such as audio files from interviews, are coded and do not contain any social security numbers that can identify the participant. Audio files are passed to the transcriber through a secure line at Karolinska Institutet.

### Confidentiality {27}

Patient data is handled in accordance with The Swedish Data Protection Act and (since May 2018) GDPR. All study-related information and participant information is stored securely at the study site trial units, with limited access. At each study site, a study participant identification log is preserved with enough information to link participants’ medical records with their study ID. This ID is used in eCRFs, on HRQoL questionnaires, and study-specific patient diaries. Participants’ study information will not be released outside of the study, except as necessary for verification of clinical study procedures by external experts bound by professional secrecy (authorised representatives from regulatory authorities and study monitors).

Ancillary studies

1. For the biomarker study, the abovementioned routines apply.
2. For the qualitative study, the study ID is used for audio files and interview transcripts to maintain confidentiality, with data securely stored at Karolinska Institute.

### Plans for collection, laboratory evaluation and storage of biological specimens for genetic or molecular analysis in this trial/future use {33}

For biobanking and analysis at the end of the ancillary biomarker study, both a test tube with whole blood (at baseline visit) and three test tubes with serum (at five visits) will be sent for storage at − 80 °C.

## Statistical methods

### Statistical methods for primary and secondary outcomes {20a}

All results will be reported according to the Consolidated Standards of Reporting Trials Guidelines, including the extension for patient-reported outcomes [59, 60].

In general, all endpoints, demographic and baseline data will be summarised using descriptive statistics and graphs as appropriate. Continuous variables will be summarised by descriptive statistics (number of patients (*n*), mean, standard deviation (SD), minimum, median and maximum). Categorical variables will be summarised in frequency tables (frequencies and percentages). Statistical tests used to compare between treatment groups will be done two-sided at a significance level of 5%, unless otherwise stated. In addition to *p* values, point estimates and corresponding 95% confidence intervals (CI) will be presented. A separate Statistical Analysis Plan will be written before unblinding the trial, giving more detailed information about the statistical analyses.

Primary endpoint is overall survival time. Overall survival time is defined as the time from date of randomisation until death. Any patient not known to have died at the time of analysis will be censored based on the last recorded date on which the patient was known to be alive, i.e. their status must be known on the censored date and should not be lost to follow-up or unknown.

A log rank test will be performed for the primary analysis of OS time. The following hypothesis will be tested:

- H0: no difference between ME and placebo
- H1: difference between ME and placebo

This log rank analysis is equivalent to the Cox proportional hazards model and will be stratified for study centre and systemic oncological treatment (not eligible for palliative chemotherapy or start of palliative chemotherapy) as strata. Stratification factors will be included in the model as covariates. Relevance of stratified analysis will be examined and if not relevant, a model without centre and/or systemic oncological therapy as strata will be used. Results will be presented in terms of an estimate of the hazard ratio (ME: placebo), associated 95% CI and *p* value. Point estimates of the median OS time will be presented for each treatment group, and OS will be displayed graphically using Kaplan-Meier plots. A per protocol analysis will be performed for OS as a sensitivity analysis. This analysis will exclude any patient who has at least one significant protocol deviation believed to have a potential impact on the efficacy outcome (OS), e.g. patients who received the wrong treatment, not enough treatment, or receiving prohibited therapy.

The secondary hypothesis to be tested regarding “global health status/quality of life (QoL)”, “physical function”, “fatigue” and “appetite loss” as scales of the EORTC QLQ- C30 questionnaire is:

- H0: no difference between ME and placebo
- H1: difference between ME and placebo

Key secondary endpoints are the corresponding scales of the questionnaire EORTC QLQ-C30.

Statistical tests for the secondary null hypothesis will take into account the bias introduced by the expected shorter follow-up time in the control arm. Details of the statistical analysis will be given in the Statistical Analysis Plan.

Other secondary endpoints are the remaining scales for HRQoL according to EORTC QLQ-C30 and EORTC QLQ-PAN-26, body weight, corticosteroid use, adverse events, costs for supportive care and for inpatient care.

#### Analysis data sets

- Full Analysis Set (FAS)—All randomised patients who received at least one dose of ME or placebo will be included in the statistical analyses of primary and secondary endpoints. FAS is equivalent to an ITT analysis set, as all participants will take their first injection with the study drug at baseline visit. To be included in the analysis of the secondary endpoints, a baseline value and at least one post baseline assessment is required. Patients will be included in the treatment groups according to randomisation. Patients lost to follow-up or withdrawing consent from the trial will be censored for the primary analysis and will not be replaced.
- Per Protocol (PP) analysis set—A per protocol analysis will be performed for OS as a sensitivity analysis. This analysis will exclude any patient who has at least one significant protocol deviation believed to have a potential impact on the efficacy outcome (OS), e.g. patients who received the wrong treatment, not enough treatment, or patients receiving prohibited therapy. Decisions regarding major protocol deviation will be made before unblinding the trial.
- Safety Analysis Set (SAS)—All randomised patients who received at least one dose of ME or placebo will be included in the statistical analyses of primary and secondary endpoints. Patients will be included in the treatment groups according to treatment actually given. SAS represents a PP analysis set including all participants that have started treatment.

### Interim analyses {21b}

No interim analysis will be performed.

### Methods for additional analyses (e.g. subgroup analyses) {20b}

Subgroup analyses will be performed regarding possible confounders such as prior and concurrent cytotoxic treatment, performance status and concomitant medication for symptom relief.

### Methods in analysis to handle protocol non-adherence and any statistical methods to handle missing data {20c}

Patients lost to follow-up or withdrawing consent from the trial will be censored at end of treatment and will not be replaced. We follow EORTC’s recommendations on calculation and handling of missing data [61] in HRQoL questionnaires. We do not plan to use imputation when reporting HRQoL data.

### Plans to give access to the full protocol, participant level-data and statistical code {31c}

The full trial protocol will be shared on reasonable request. Anonymised data on group level may be shared with scientists who have medically or scientifically well-founded reasons; data protection according to GDPR and ethics according to ethical approval must be ensured.

## Oversight and monitoring

### Composition of the coordinating Centre and trial steering committee {5d}

#### Trial steering

Trial design and study protocol, critical review trial-related documents, supervision of trial organisation and conduct of trial.

#### Data manager

Design of and support of randomisation and eCRF system, study drug distribution.

#### Coordinating investigator

Design, study protocol and revisions, application/amendments to medical drug agency and ethics, trial registration, preparation of trial-related documents, organisation of national trial meetings, newsletter, annual safety reports, review severe adverse event (SAE) reports.

#### Principle investigators ancillary studies

Study design, protocol, related trial documents and conduct of studies.

#### Principle investigators and study nurses at study sites

Principal investigator takes responsibility for supervision of the trial at each study centre and ensures compliance with study protocol.

Assigned study nurses ensure follow-up according to protocol and delegations

### Composition of the data monitoring committee, its role and reporting structure {21a}

A data safety committee regularly reviews SAE reports and decides upon discontinuation of the trial in the event of severe delay of recruitment or severe quality deficiencies.

The trial is monitored by an experienced and independent monitor. First monitoring is performed after the first inclusions, and thereafter at least once per year. Study sites with high inclusion rates are monitored at least twice per year.

### Adverse event reporting and harms {22}

In this study, an adverse event is defined as any untoward medical occurrence in a participant without regard to the possibility of a causal relationship.

Expected events such as symptoms from disease or known side effects form oncological or surgical treatment or from the study drug are *not* regarded as AE. Examples for expected events are progression of malignancy, including fatal outcome, laboratory deterioration, hospitalisation due to malignancy progress/symptoms as they are expected due to the nature of a progressing disease and planned hospital visits such as for chemotherapy treatment. Other possible expected events are local reactions and overreactions at injection site (not regarded as AE but documented in the eCRF), increased body temperature ≤ 38C, and treatment failure of the study drug.

Unexpected events are to be reported as AE. In case of any doubt, investigators are instructed to report.

If an adverse event is considered serious, severity and possible relationship to study drug are defined and the SAE is reported by fax to the coordinating investigator at the sponsor site via a SAE form; a final report is sent when the SAE is resolved. Suspected Unexpected Serious Adverse Reactions (SUSARs) are reported by the coordinating investigator (or designee) to the Swedish Medical Product Agency within 24 h of knowledge or at the latest on the following working day.

### Frequency and plans for auditing trial conduct {23}

No audits are planned because this trial is academic.

### Plans for communicating important protocol amendments to relevant parties (e.g. trial participants, ethical committees) {25}

According to national regulations, major modifications of the protocol require a formal amendment to the protocol and are to be approved by relevant parties (Swedish Ethical Review Authority, Medical Drug Agency) and communicated to participating study sites.

### Dissemination plans {31a}

The trial results will be submitted for publication in relevant medical journals with authorship stated according to the requirements for manuscripts in the Vancouver Statements.

## Discussion

Considering the pessimistic prognosis with short expected survival and considerable symptom burden for patients with advanced pancreatic cancer, there is an urgent need for more effective treatment options to prolong OS and improve HRQoL. It is of uttermost importance to find tolerable and potent therapies for the large proportion of newly diagnosed patients with advanced disease and frail performance status who currently have no treatment options other than best supportive care. Similarly, improved palliative treatments are necessary for patients with recurrent disease following curative intent treatments, as well as for patients with advanced disease and preserved performance status who are eligible for chemotherapy but have limited benefit from the latter. To our knowledge, this is the first placebo-controlled randomised trial assessing the impact of ME on OS and HRQoL in patients with advanced and/or relapsing pancreatic cancer. A previous randomised study from Serbia suggests that ME prolongs OS and increases HRQoL [35, 36], but the lack of placebo control and the exclusion of patients with palliative chemotherapy make generalisation difficult. The present trial was designed to overcome these limitations by adding a placebo group and including patients with and without chemotherapy. In the light of a recently established, international core set of patient-reported outcomes [62] such as global health status/QoL, physical ability, ability to work/do usual activities and abdominal complaints, our choice of secondary outcomes is highly clinically relevant. Moreover, the advanced integration of palliative care in the Swedish health care system [63] and the addition of two ancillary studies on biomarkers and qualitative interviews may contribute with other baseline data and in vivo effects of controlled ME, as compared to the study conducted in Serbia.

In the light of the increasing role of the immune system in oncological treatment development, it is essential to further investigate this relatively non-toxic immunomodulating therapy. As there is no previous connection of OS and HRQoL to immunological response, the biomarker study in a placebo-controlled double-blind setting may enable robust identification of ME-specific predictive biomarkers, as well as general prognostic biomarkers.

Many cancer patients use and value complementary therapies [64] including ME preparations [12, 14, 65] and express a wish for evidence and professional guidance in their decisions—a precondition for patient safety and satisfaction. Systematic research is needed to enable professional guidance.

The sample size of this trial was pragmatically chosen and based on previous observed effects of ME [35, 36] as well as practical circumstances and available funding. Given the short survival in this type of cohort and therefore short time until mature data are obtained, and the reasonably rapid inclusion of new patients, an interim analysis was not considered suitable.

Previous randomised trials on ME in Germany, a country with high popularity and high use of ME in general health care, have shown slower recruitment of patients with advanced stages of cancer and frequent prior ME treatment [66], as well as difficulties of enrolment and randomisation exceeding trials in conventional oncology. This is likely to be partially caused by the widespread knowledge, use and popularity of ME in Germany [67]. Sweden is a country with low usage and rare prescription of ME [65] and should therefore provide a more feasible study environment for recruitment and retainment of participants in a placebo-controlled ME trial design.

Generally, trials in oncology are often difficult to blind, since the active treatment, e.g. chemotherapy or immunotherapy, confers obvious adverse events and complications which would not occur in a placebo arm. To date, there is no placebo capable of exactly mimicking the possible local reactions such as they might occur from ME at higher dosages without conferring a risk of toxicity. Local reactions have been shown to be more common in females, younger patients, during chemotherapy and in patients with lower tumour stage [48]. Our study population consists of patients of both sexes in advanced tumour stage and many participants being treated with chemotherapy. Subcutaneous injections in general may cause non-specific local redness immediately after injection. While the patients receive general information about local redness following any type of subcutaneous injection, no specific remarks are made on potential local reactions specific to ME. We therefore expect successful blinding in all control patients and in most patients of the treatment arm. Nevertheless, all reported local reactions and local overreactions are documented systematically to enable an estimation of potential unblinding. In any case, a significant effect on OS by expecting just another medication in such a severe disease as advanced pancreatic cancer is highly unlikely [68]. Even regarding HRQoL, expectation effects are questionable in cancer patients [69].

Two more effective chemotherapy combinations (gemcitabine/nab-paclitaxel and FOLFIRINOX) have been introduced at about the same time as trial start, resulting in longer OS (about 8 months instead of 5 months) for a smaller group of highly selected patients eligible for these treatments. We chose not to modify the study protocol and insert another stratum apart from the original chemotherapy/no chemotherapy stratification. In the unlikely event of unequal distribution of this variable, this will be accounted for through regression analysis.

The biomarker study provides a unique opportunity to assess mechanisms of action of ME in vivo in a randomised controlled setup. ME-induced implications on blood cell counts including leucocyte subtypes, cytokine and immunoglobin levels, anti-viscotoxin and-mistletoe lectin antibodies and other serological components will be evaluated, as well as potentially prognostic and treatment predictive baseline parameters in blood/serum. DNA isolated from peripheral leucocytes may be analysed in terms of germline sequence variations and their relation to the outcome on ME in advanced pancreatic cancer.

The qualitative study is expected to complement the trial results with data on participants’ own experiences such as symptoms, every-day life during the palliative phase of pancreatic cancer and participation in a placebo-controlled study with ME. The analysis of this data may extend, clarify and increase the understanding of unexpected or equivocal data generated from the trial.

In summary, the presented trial with its two nested ancillary studies is expected to give new insights in the treatment of advanced pancreatic cancer.

## Trial status

The current protocol version is 3.3 (date 15 July 2020). This trial opened for recruitment on 1st June 2016 and is expected to complete recruitment by 2021.

## Data Availability

Any material required to support the protocol can be supplied on reasonable request.

## Abbreviations

AE: Adverse event
CRF: Case report form
CTCAE: Common Terminology Criteria for Adverse Events
ECOG: Eastern Cooperative Oncology Group
eCRF: Electronic case report form
EORTC: European Organisation for Research and Treatment of Cancer
GCP: Good Clinical Practice
GDPR: General Data Protection Regulation
HRQoL: Health-related quality of life
ME: Mistletoe extract
OS: Overall survival
QoL: Quality of life
SAE: Severe adverse event
